# Endoscopic ultrasound-guided modification of surgical anatomy (from Roux-en-Y gastrectomy to Billroth II-like anatomy) for endoscopic treatment of malignant biliary stenosis

**DOI:** 10.1055/a-2095-2267

**Published:** 2023-06-22

**Authors:** Giulia Piazza, Enrique Lázaro-Fontanet, Arthur Cotton, Sébastien Godat, Domenico Galasso

**Affiliations:** 1 Surgical Department, Hôpital Riviera-Chablais, Rennaz, Switzerland; 2 Gastroenterology and Hepatology Department, University Hospital CHUV, Lausanne, Switzerland; 3 Gastroenterology Department, Hôpital Riviera-Chablais, Rennaz, Switzerland

We describe the case of an 84-year-old man who presented with obstructive jaundice. Seventeen months prior to this, he underwent a Roux-en-Y gastrectomy for gastric adenocarcinoma (pT4a pN3a pM1 (peritoneal carcinosis)) and was being treated with pembrolizumab.

At the time of presentation, a computed tomography (CT) scan and magnetic resonance imaging showed a 24-mm nodule at the gastric stump and a 9-mm common bile duct stricture causing upstream dilatation ( Fig. 1 ). This was corroborated with the findings of previous positron emission tomography (PET)-CT done 2 months before, which showed a lesion of hypermetabolic activity at the duodenal stump ( Fig. 1 ).

**Fig. 1:**
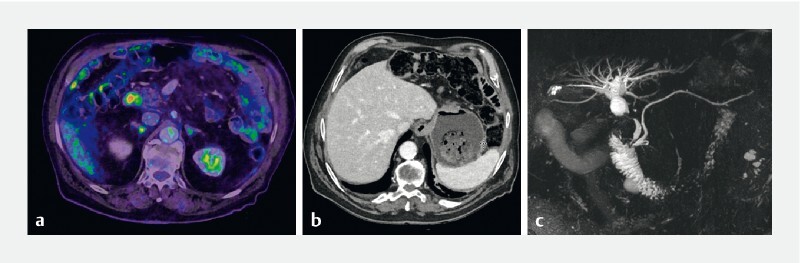
Pre-treatment cross-sectional imaging of the patient. **a** Positron emission tomography-computed tomography showing non-specific hypermetabolic activity at the duodenal stump. **b** Computed tomography scan highlighting the presence of a 24-mm nodule at the gastric stump. **c** An indeterminate common bile duct stricture, 9 mm long, causing upstream dilation on magnetic resonance cholangiopancreatography.

With the patient’s consent, we decided to create a communication between the gastric stump and the duodenum, in a Billroth II-like anatomy. This option was inspired by publications from other centers 1 2 3 4 5 and provided considerable advantages. The duodenal stump could be visualized, repeated endoscopic retrograde cholangiopancreatography (ERCP) could be performed as well as a safety net created should any recurrence at the gastro-jejunal anastomosis occur.

During the first procedure, in which a communication between the gastric stump and the duodenum was created, a biopsy of the nodular lesion at the gastric stump was performed and confirmed adenocarcinoma recurrence. Two days later, the patient had an ERCP to facilitate biliary drainage as well as a biopsy of the biliary stricture and duodenal stump. A third procedure became necessary due to an ongoing cholestatic picture and the onset of cholecystitis in which the gallbladder was drained.

The described technique allowed us to repeat the ERCP to remove gallstones lodged in the biliary stent as well as to perform further endoscopic procedures in the future where necessary.

The patient had an uneventful recovery, with his liver function tests normalizing within 1 month, and he was able to restart his chemotherapy.

The video ( Video 1 ) shows the four procedures and the final result.

**Video 1** Modification of surgical anatomy from Roux-en-Y gastrectomy to Billroth II-like anatomy for endoscopic treatment of malignant biliary stenosis.


Endoscopy_UCTN_Code_TTT_1AS_2AD

## Notes

Endoscopy E-Videos

https://eref.thieme.de/e-videos
*E-Videos* is an open access online section of the journal *Endoscopy* , reporting on interesting cases and new techniques in gastroenterological endoscopy. All papers include a high-quality video and are published with a Creative Commons CC-BY license. Endoscopy E-Videos qualify for HINARI discounts and waivers and eligibility is automatically checked during the submission process. We grant 100% waivers to articles whose corresponding authors are based in Group A countries and 50% waivers to those who are based in Group B countries as classified by Research4Life (see: https://www.research4life.org/access/eligibility/ ). This section has its own submission website at https://mc.manuscriptcentral.com/e-videos
